# Post-diagnostic support for adults diagnosed with autism in adulthood in the UK: A systematic review with narrative synthesis

**DOI:** 10.1177/13623613241273073

**Published:** 2024-09-10

**Authors:** Jade Eloise Norris, Rebecca Harvey, Laura Hull

**Affiliations:** University of Bristol, UK

**Keywords:** adulthood, autism, diagnosis, peer support, post-diagnostic support, psychoeducation

## Abstract

Autism diagnoses in adulthood have risen substantially over recent years. While providing potential benefits, many autistic adults struggle to navigate their new diagnosis. Our review aimed to synthesise the evidence for post-diagnostic support for autistic adults without intellectual disability diagnosed in adulthood in the UK: investigating availability, efficacy, and autistic adults’ experiences. A systematic search of four biomedical and three grey literature databases was conducted to identify UK-based studies published since 2012. A total of 27,581 records were screened in duplicate, with 19 studies regarding post-diagnostic support included. A narrative synthesis was undertaken due to anticipated heterogeneity. A service mapping exercise also provided a summary of post-diagnostic service provision currently available across the UK. Findings indicate that many services across the UK provide information and signposting as their primary form of post-diagnostic support, whereas autistic adults desire low-level support services, psychoeducation, and peer support. Evidence regarding the efficacy of post-diagnostic support was unavailable. This review highlights the need for adequate support to alleviate post-diagnostic challenges and improve autistic adults’ quality of life. Low-level support services, psychoeducation, and peer support appear acceptable and feasible, with a preference often indicated for autistic-led support. Future research should develop manualised post-diagnostic support programmes and assess their efficacy.

**Lay abstract:**

More adults than ever before are seeking an autism diagnosis in adulthood. While receiving a diagnosis may be beneficial, many autistic people struggle to navigate their new diagnosis, and require support. This study conducted a systematic review of previous research on the support available after diagnosis (post-diagnostic support) for autistic adults without intellectual disability who were diagnosed in adulthood in the UK. A systematic review is a pre-planned method of searching for all relevant studies, before combining these to answer a larger question. The study aimed to investigate the availability of such support and its effectiveness, and to explore autistic adults’ experiences of accessing support. We also used publicly available information to create a map of the post-diagnostic support services currently available across the UK. A systematic search of seven databases was conducted, to identify UK-based studies published after 2012. Nineteen studies were eligible to be included in the study. Although some form of post-diagnostic support is available across most areas in the UK, this mostly consists of providing information and ‘signposting’ the person to other services. These options may not meet the needs of autistic people, who want services such as psychoeducation (therapy whereby an individual receives education about their diagnosis to improve understanding and self-management), and peer support. Findings highlight the need for adequate support to alleviate the post-diagnostic challenges autistic adults face. The study could not evaluate the effectiveness of support options in the UK due to a lack of information about this in published research. Research shows that autistic adults would like low-level support services, psychoeducation, and peer support, and may also prefer autistic-led support. Further research is required to develop and evaluate post-diagnostic support programmes which include these elements.

Autism spectrum disorder (ASD), while complex and heterogenous in presentation, is characterised by core difficulties in communication and social interaction, as well as restricted or repetitive behaviours, and presents with or without intellectual disability.^1^ Although lifelong, and historically thought of as a diagnosis of childhood, it is increasingly acknowledged that autism may be unrecognised or misdiagnosed, resulting in presentation for diagnosis later in life (Au-Yeung et al., 2018; Punshon et al., 2009).

The prevalence of autism in the UK is relatively high (1.1%; National Institute for Health and Care Excellence, 2020). Diagnoses have risen exponentially by 787% from 1998 to 2018, with the greatest rise for any age group seen in adults (Russell et al., 2021). This increase is likely due to heightened awareness and improved diagnostic services following the introduction of the first disability-specific legislation in England; the Autism Act (2009), and the first Autism Strategy in 2010 (National Autistic Society, 2020).

Receiving a diagnosis has been credited with helping autistic adults to gain a better understanding of themselves, bringing a sense of relief (Powell & Acker, 2015; Stagg & Belcher, 2019), and may lead to improvements in mental health (Arnold et al., 2023). However, for some, it represents a shock, and can trigger negative emotions (Powell & Acker, 2015). Even when viewed positively, many autistic adults can struggle after their diagnosis. Difficulties include re-aligning their self-identity (revisiting potentially distressing past experiences during the diagnostic process), feelings of isolation, including the potential for discrimination and negative reactions from others, and impacts upon mental health (Leedham et al., 2020; Punshon et al., 2009; Stagg & Belcher, 2019). This is magnified by the fact that autistic individuals already face poorer health outcomes, with significantly higher rates of mental health disorders (Lai et al., 2019).

Given the substantial difficulties experienced by many autistic adults, support in this post-diagnostic period is vital. Post-diagnostic support can assume many different forms, including (but not limited to) the provision of information and signposting to other services; low-level support services to assist in the management of day-to-day life; peer support; and psychoeducation. Psychoeducation describes a structured therapeutic intervention involving education on a condition in order to improve understanding and self-management, delivered individually or in groups (Sarkhel et al., 2020). It has been more widely evaluated for other conditions, for example, for attention deficit hyperactivity disorder (ADHD), where psychoeducation has demonstrated efficacy in increasing knowledge of the condition and improving life satisfaction (Hirvikoski et al., 2017). The efficacy of psychoeducation or other support options for autistic adults is still unknown.

Many autistic adults have reported that support following their diagnosis is lacking or inaccessible, particularly for those without intellectual disabilities (Arnold et al., 2023; Powell & Acker, 2015; Public Health England, 2017; Stagg & Belcher, 2019). If no meaningful support is available following receipt of a diagnosis, the purpose of diagnostic services is limited. Within the UK, most post-diagnostic support is provided through the publicly-funded (i.e., free at the point of use) National Health Service (NHS), although this can include commissioning or paying for support offered by organisations and healthcare providers external to the NHS. Often due to long waiting times for such services, some adults may also choose to pay for private post-diagnostic support, although very little information is available on the prevalence or quality of such support.

The most recent UK Autism Strategy (2021) promises funding to improve post-diagnostic pathways (Department of Health and Social Care and Department for Education, 2021). However, there is a lack of evidence regarding current post-diagnostic support for autistic adults without intellectual disabilities diagnosed in adulthood. To address this gap, our review aims to investigate the current provision of post-diagnostic support for these adults in the UK, assess the efficacy of post-diagnostic support, and explore autistic adults’ experiences with post-diagnostic support. Through this, we aim to inform the design and commissioning of adult post-diagnostic services.

## Methods

### Overview

**Figure 1.**
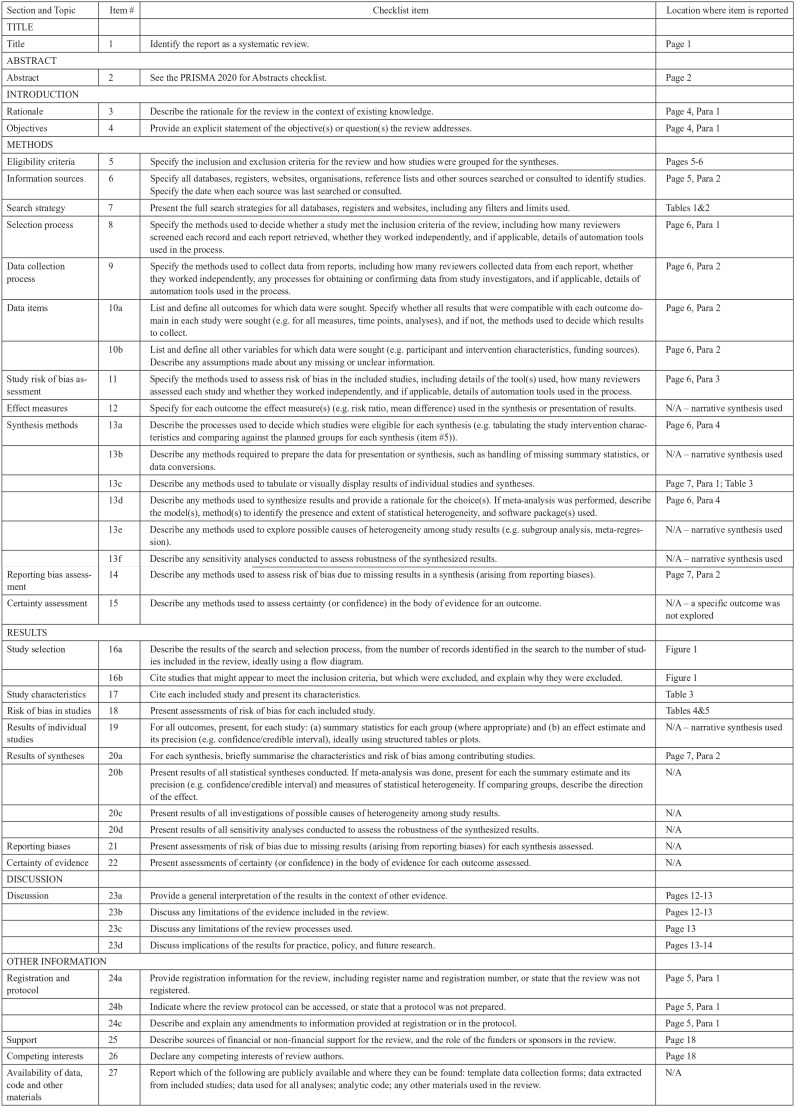
PRISMA checklist. Source: Page et al. (2021).

A protocol was registered with PROSPERO, the online International Prospective Register of Systematic Reviews (registration #CRD42022336677). The inclusion criteria were amended to incorporate studies with participants aged 16+ years with at least some diagnosed in adulthood, due to a paucity of studies identified in the initial search; otherwise, the review proceeded as intended. The review followed the Preferred Reporting Items for Systematic Reviews and Meta-Analysis (PRISMA) guidelines for conducting systematic reviews (Page et al., 2021; see Figure 1).

### Search strategy

A systematic search of four biomedical databases was conducted on 16 June 2022: MEDLINE, PsycINFO, CINAHL and Web of Science. A comprehensive search strategy was developed by consulting topic literature (Supplemental Materials A and B). Due to varying methods of post-diagnostic support and an anticipated lack of studies, we aimed for a sensitive search and tolerated low specificity to ensure we captured all relevant studies. We restricted the search to English language studies published since 2012 to identify evidence relevant to current practice. Searches were re-run on 16 July 2022, and 10 April 2024 to locate any additional eligible studies. Citation searching (forwards and backwards) was also utilised.

Grey literature searches of Google Scholar, EThOS, and ProQuest were conducted for unpublished literature, allowing a richer evidence base. In addition, online searches were conducted on Government, NHS, Clinical Commissioning Group, Integrated Care System, and Council websites to identify other eligible studies.

### Selection criteria

The inclusion criteria were:

1. Primary research studies of any design;
2. Involving adults diagnosed with autism, without intellectual disability, in adulthood (note that studies were eligible if they featured some adults diagnosed in childhood);
3. Regarding post-diagnostic support of any form;
4. and UK-based.

The exclusion criteria were:

1. Studies involving only children or only adults diagnosed in childhood, or adults diagnosed with autism and an intellectual disability;
2. Regarding interventions not utilised as, or available as, post-diagnostic support;
3. and non-UK based.

### Screening and full-text review

**Figure 2.**
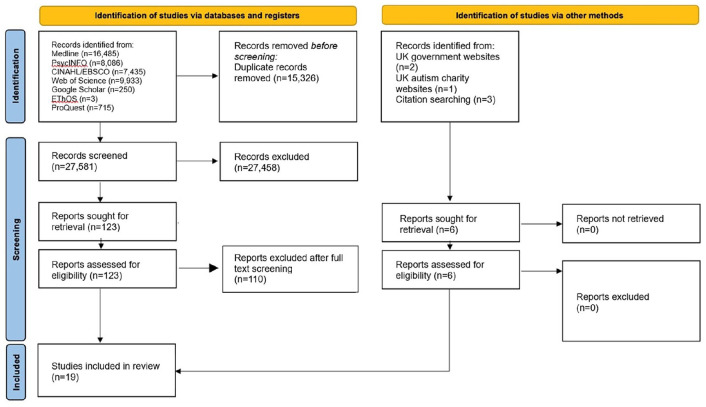
Flow of studies through the systematic review process.

Duplicate references were removed automatically using EndNote 20, and the remaining references were manually checked for missed duplicates and then exported into the screening software system Rayyan (Ouzzani et al., 2016). Titles and abstracts were independently double-screened, with any conflicts resolved by discussion. The second author then completed the full-text review for the first two searches, with the first and last author completing full-text review for the final search. The flow of studies is displayed in Figure 2.

### Data extraction

Extracted data and quality assessment findings were imputed into Microsoft Excel (2022) by the second author, using a standardised form created for this review. Extracted information comprised the study aim; year; location; description of post-diagnostic support if applicable; study design; methodology; participants; inclusion/exclusion criteria; sampling; recruitment and retention; key findings; funding and conflict of interest; and strengths and limitations.

### Quality assessment

The quality of journal articles was assessed using the Mixed Methods Appraisal Tool (MMAT), chosen for its application to diverse study designs (as anticipated with this review), and its domain-based (rather than score-based) approach (Hong et al., 2018; see Supplemental Material C). Grey literature quality was assessed using the Authority, Accuracy, Coverage, Objectivity, Date Significance (AACODS) checklist, selected for its comprehensive nature and domain-based approach (Tyndall, 2010; see Supplemental Material D).

### Evidence synthesis

A narrative synthesis approach was chosen, due to the heterogeneity of study designs and aims which rendered meta-analysis unfeasible. Synthesis followed the framework provided by Popay et al. (2006) and the Synthesis Without Meta-analysis (SWiM) reporting guidelines (Campbell et al., 2020; Popay et al., 2006). Studies were grouped for synthesis according to the three components of our review aim, and sub-grouped by the post-diagnostic support method. We prioritised studies according to the volume of data regarding post-diagnostic support, and the quality assessment findings.

### Service Mapping

A secondary aim of the study was to provide an overview of the currently available post-diagnostic support for autistic adults in the UK. All services identified through the systematic review were included in this mapping exercise, as well as services identified on Government, NHS, Clinical Commissioning Group, Integrated Care System, and Council websites which did not meet inclusion criteria for the systematic review; for instance, services which were described online only but were not referred to in any research studies.

## Results

We included 19 studies in this review: 13 journal articles and 6 grey literature reports (summarised in Table 1). These were of varying study designs (8 qualitative, 4 quantitative and 7 mixed methods), represented all four UK countries and were published between 2014 and 2024. Below, we present summaries of the quality assessment, and characteristics of the participants to better describe the sample. We then present the findings related to (a) efficacy of post-diagnostic support options, (b) experiences of participants and (c) mapping the available services identified.

**Table 1.** Summary of included studies.

First author (year)	Study type Location Participants^a^	Aim Study design Methodology	Key findings relating to post-diagnostic support^b^	Key limitations
Hull et al. (2024)	**Study type**: Journal article **Location**: UK **Participants**: 11 autistic adults	**Aim:** To interview autistic adults about their past experiences of Social Skills Training (SST), and their perceptions regarding how such support could be improved. **Design:** Qualitative **Data collection:** 1:1 semi-structured interviews (online; video call (with option for audio only); or responses could be typed into the chat box)	Participants shared recommendations for factors which make SST effective and enjoyable. In particular, the participants valued interactive SST, which utilised, for example, roleplay and videos, as these methods may be more engaging than, for example, lecture-style information delivery. Interviewees also emphasised the importance of activities being relevant to their lives, with some participants valuing the opportunity to practise SST-related tasks in a ‘safe’ environment. Satisfaction with SST was also influenced by the facilitator, who was, in most cases, a non-autistic adult. Participants recommended that such training could be designed and delivered by autistic facilitators. The interviewees also emphasised that SST should be personalised to the participants, including granting autonomy to determine specific areas where support was desired (i.e., a person-centred approach). The clinical context in which SST takes place was also noted as being important; the majority of the interviewees had taken part in SST whilst also learning about their (often new) autism diagnosis, and with some also managing co-occurring mental and physical health problems. In addition, the interviewees reported some negative experiences of SST, including feeling that SST had reinforced the stigma that their autism was ‘bad’ or ‘wrong’, with some participants also suggesting that using what they may consider inauthentic social skills learnt via such interventions may lead to masking or camouflaging their autistic characteristics, which may have negative consequences for their mental health.	Self-reported data which may be affected by biased remembering; relatively small and majority White sample.
Crowson et al. (2024)	**Study type**: Journal article **Location**: UK **Participants**: 43 and 139 autistic adults	**Aim:** To establish the priorities of autistic adults for optimal provision of post-diagnostic support. **Design:** Modified Delphi study **Data collection:** A series of three online questionnaires with 43 autistic adults to identify support priorities, followed by a fourth questionnaire completed by 139 autistic adults to identify consensus on priorities for post-diagnostic support.	24 priorities met or exceeded the stated consensus threshold of 80% of participants agreeing/strongly agreeing that these items represented important elements wished for in post-diagnostic support. Participants prioritised access to support in their area, adequate training of professionals, support with processing the impact of a diagnosis in adulthood, consistency in use of their preferred methods of contact, and the provision of an individualised support plan.	Relatively small sample sizes per Delphi round; diversity of sample
Beresford & Mukherjee (2023)	**Study type:** Report to NHS England **Location:** UK**Participants:**26 autistic adults; 25 practitioners; 5 experts by experience; 4 family members	**Aim:** To further understand the information and support needs arising when being diagnosed with autism as an adult; to identify interventions and practices required to meet these needs **Design:** Consultation work (with autistic adults, practitioners, and the wider autism community); review of existing autism psychoeducation programmes **Data collection:** Consultation: Focus groups, and 1:1 conversations and workshops Review: systematic collation of information about services currently being offered, and reviewing this against findings from the consultation work	Participants noted intense emotional responses to receiving an autism diagnosis in adulthood, as well as wanting help with navigating their questions regarding what this new diagnosis may mean for them, and how the process of diagnosis, and in particular the focus on deficits during the process, was often difficult. In addition, participants noted the importance of receiving not only confirmation of their difficulties but also suggested solutions (i.e., meeting their post-diagnostic needs), and also meeting the needs of their families/carers. Consultation participants felt strongly that responding to the psychoeducational needs of adults receiving an autism diagnosis requires a multi-faceted approach, and the need for flexibility (e.g. in approach, such as 1:1 delivery vs group-delivered sessions), as well as adequate resources and capacity to enable personalised psychoeducation. Meeting psychoeducation needs: respondents felt that services providing diagnosis should also provide post-diagnosis psychoeducation about autism, alongside support with healthcare, social and other needs. Autistic people also emphasised that in the design and delivery of an effective and acceptable psychoeducation programme, the following were important factors: choice of autism practitioner and the involvement of experts by experience; the content, structure and approach; size of the group and its make-up; timing and venue and mode of delivery.	Non–peer-reviewed publication
Crane et al. (2023)	**Study type**: Journal article **Location**: UK **Participants**: 16 autistic adults	**Aim**: To replicate previous research (Crane et al., 2021; see below) to determine whether similar results were obtained when the *Exploring Being Autistic* programme was delivered online as opposed to in-person **Design**: Qualitative **Data collection**: Questionnaire and post-programme (within a group or 1:1) interviews (one upon completion of the programme, and another 6–8 months later) **Data analysis**: Reflexive thematic analysis	Participants reported positive experiences of participating in the programme, in particular, that it was autistic-led. Participants developed a positive and practical outlook after attending the programme. There were mixed views regarding online delivery, with some noting that remote participation acted to reduce perceived cognitive load, which meant that it was accessible to more participants, allowing the development of meaningful social connections amongst the participants. However, some technological and practical issues caused barriers, and some felt that aspects of participation, such as breakout groups, didn’t work as well.	Some attendees not completing the second interview (possible self-selection bias)
Crompton et al. (2022)	**Study type**: Journal article **Location**: UK **Participants**: 12 autistic adults	**Aim**: To explore the views of those receiving an autism diagnosis in adulthood, their diagnostic experiences, and post-diagnostic support needed and provided **Design**: Qualitative **Data collection**: 1:1 semi-structured interviews (online, face-to-face, or telephone) **Data analysis**: Thematic analysis	Four themes identified: ‘mismatch in support needed and provided’, ‘community connection’, ‘flexible and personalised support’, and ‘sustainability’. Post-diagnostic support did not meet participants’ needs. Most information was perceived as insufficient, unhelpful, or inappropriate. Participants did not want information about autism, but wanted to understand how their autism diagnosis applied to their lives. Many found it difficult to reach out to services due to communication difficulties. All respondents described difficulties processing the diagnosis and reframing their history, and wished for help with this. Many experienced negative emotions post-diagnostically, and lacked acces to an understanding person to discuss this with. Many wanted support with building a sense of identity and community, as well as self-care skills and coping strategies. Instead, they were left feeling ‘unsure how to manage this’. Furthermore, many who requested post-diagnostic support were told none was available or were refused. Participants wished for connections with like-minded peers and a community. Most felt that spending time with other autistic people in a peer support setting would have been beneficial in understanding oneself and validating their experiences. Participants felt that peer support may offer the flexibility needed to improve their post-diagnostic experience. Relationships with other autistic individuals were easier and more comfortable, with less need to mask. Participant preference was for autistic-led peer support. However, it was noted that autistic people are not solely defined by autism, and peer support should consider other aspects of their backgrounds. The peer-support framework should be flexible and responsive to the needs of group members. Participants expressed a need for peer support to be manageable and sustainable. Facilitators should be trained or experienced and supervised, and simply being autistic does not provide someone with the skills or knowledge required. Participants noted that support may be required from other services, including mental health services, and that peer support should not be used in place of specialist support, but work alongside it.	Representativeness; self-selecting sample; variable previous exposure to peer support
Minister for Mental Wellbeing and Social Care (2022)	**Study type**: Grey literature government report **Location**: Scotland **Participants**: 99 autistic adults	**Aim**: To evaluate the national post-diagnostic support service **Design**: Quantitative **Data collection**: Online survey with closed-format questions (developed using an expert panel and Delphi process) **Data analysis**: Descriptive statistics **NB**: Qualitative data collected from free text boxes and presented as quotes, but not analysed	Data were aggregated for all autistic adults and not divided into which specific post-diagnostic support was experienced. Most autistic adults felt the information about the service they used was clear and accessible (79%), and felt listened to by the service (89%), with clear advice provided (82%). A majority felt their expectations were fully met or exceeded (62%). Most felt the service provided good information about opportunities and signposting to other services (75%). Almost all would recommend the post-diagnostic service to others (93%). Almost all autistic adults surveyed felt they had a better understanding of autism since receiving post-diagnostic support (90%). Most felt it enabled them to know and understand themselves better (87%). The majority felt they now had a connection with other autistic peers (67%). Other improvements noticed by some participants were improved positivity/self-esteem (59%), improved confidence (52%), improved mental health (44%), reduced social isolation (43%), reduced anxiety (41%), improved skills in coping with uncertainty (31%), reduced stress (29%), and improved general health (21%). Qualitative comments suggested a more positive experience of the post-diagnostic phase than the diagnostic process. Support from other autistic people was significant in reducing feelings of being an ‘outsider’; finally feeling comfortable, understood, and ‘a part of something’, in many instances, for the first time; and respondents noted appreciating meeting other adults diagnosed in adulthood.	Which support each participant received unknown; breakdown of participant characteristics not available for all adults; descriptive statistics only (no other statistical methodology)
Wigham et al. (2022)	**Study type**: Journal article **Location**: UK **Participants**: 343 autistic adults	**Aim**: To explore the perspectives of autistic adults, thier relatives, and professionals regarding UK adult autism post-diagnostic support and service provision, and to use this information to agree consensus statements for optimal post-diagnostic support services **Design**: Quantitative **Data collection**: Online or paper survey with closed-format questions (developed by the research team, and informed by international clinical guidelines) **Data analysis**: Descriptive statistics, non-parametric analysis (Mann-Whitney and Chi-Square) **NB**: Mixed methods study; qualitative data not yet published	A small majority of autistic adults received a follow-up appointment (53.1%); 40.5% received this <12 weeks after diagnosis. At most, one-third of autistic adults reported receiving any of the types of support listed in the survey during the 12 months post-diagnosis. Services or support received: mental health support (34.1%); support with stress and/or anxiety (27.7%); financial support (25.4%); social groups (23.6%); employment support (17.5%); healthcare support (17.5%); taught groups to develop skills (9.3%). 11 consensus statements were generated following survey responses and agreement with autism experts. In summary, these statements recommended the need for all adults to receive a follow-up meeting 2–4 months after the feedback appointment post-diagnosis; a clear pathway for accessing local post-diagnostic support across levels of care, with local champions to facilitate implementation; dedicated post-diagnostic support services should be coordinated with diagnostic services but commissioned separately; expert, multi-disciplinary services which include occupational and speech and language therapy; support should be available 1:1 and in group format according to individual need; diagnosis should not limit access to mental health services; post-diagnostic services should provide step-on, step-off support.	Representativeness; closed-questions relating to the receipt of support, no ascertainment of whether other support offered or desired (though open-response questions allowed this opportunity, these data are not yet published)
Hatton & Lee (2022)	**Study type**: Journal article **Location**: UK **Participants**: 14 autistic adults **Service evaluated**: 7-week online post-diagnostic group psychoeducation programme delivered by Gloucestershire NHS Health and Care Trust; non-autistic-led	**Aim**: To evaluate a post-diagnostic group psychoeducation programme, and whether autistic clients prefer online or face-to-face groups **Design**: Qualitative **Data collection**: 1:1 semi-structured interviews (online or telephone) **Data analysis**: Thematic analysis	Four themes identified: ‘autistic community’, ‘experience of being part of an online group’, ‘opportunity for consolidation’, and ‘design considerations and improvements’. The programme was overall positively received by participants. Participants gained a sense of community from the group. They appreciated knowledge-sharing and the opportunity for self-reflection, and for consolidating the knowledge gained from the assessment. This enabled them to gain insights into how to manage day-to-day activities; subsequently, participants reported that the programme allowed them to ‘manage rather than struggle’. A sense of belonging was gained from meeting others recently diagnosed. Participants felt the group should be offered the choice of face-to-face (allowing more interaction) or online (for those who feel less anxious online, or for practicality/accessibility). Some reported difficulties with an online platform, regarding issues such as interference. Opinions differed regarding session duration and the number of attendees. Participants felt more focus should be given to the positive aspects of autism and strengths of autistic individuals, not only the negatives; and that workplace functioning could be focused on more, or additional reading provided.	Small sample; timing of interviews (immediately after completion), thus no long-term data; role of interviewer (also conducted the groups) though criticisms were discussed
Crane et al. (2021)	**Study type**: Journal article **Location**: England **Participants**: 16 autistic adults; subsample of 11 re-interviewed **Service evaluated**: 10-week in-person group psychoeducation programme *(Exploring Being Autistic)* delivered by a third-sector organisation; autistic-led	**Aim**: To evaluate *‘Exploring Being Autistic’*; to identify any benefits of the programme and ways to make it more acceptable to future participants **Design**: Qualitative **Data collection**: Pre-programme questionnaires; 1:1 in-depth interviews conducted immediately post-completion and 6 months afterwards (face-to-face or via telephone) **Data analysis**: Thematic analysis (data combined from both sets of interviews due to overlapping themes)	Three themes identified: ‘appreciation of the autistic-led nature of the programme’, ‘unity in diversity’, and ‘developing a positive, practical outlook on autism’. Participants were largely positive about the programme. They felt through learning from the experiences of others, alongside the educational components, that they were more positive about day-to-day life, more self-aware, and able to develop coping strategies that were easily utilisable in difficult situations. They reported now being able to ‘see what’s happening and find a way out of it’. One participant reported being able to mask less often subsequently. Participants felt a sense of belonging and of being less socially isolated. Sharing with others was felt to be even more important than the structured educational components. Many highlighted the autistic-led factor as a strength of the programme. Whilst many would have been accepting of a neurotypical-led programme, they preferred autistic-led. The autistic facilitator was felt to be more tolerant and understanding, and provided a positive role model for participants who had recently been diagnosed. Motivations for attending were explored. A common motivating factor was empowerment and a desire to feel accepted by meeting ‘like-minded people’, and wanting to ‘explore the positive aspects of autism’, alongside developing practical strategies and coping mechanisms.	Small sample; role of facilitator in co-designing the evaluation (though interviewer independent); replicability of programme as not manualised; difficulty attributing effect to programme or facilitator
McConkey et al. (2021)	**Study type**: Journal article **Location**: Northern Ireland **Participants**: 54 autistic adults used the service over 3 years; a subsample of 27 autistic adults represented in the evaluation **Service evaluated**: 1:1 low-level support service, ‘RIGHT4U’, commissioned for post-diagnostic support. Adults are supported to gradually increase social and employment activities by a trained support worker, with peer support subsequently introduced and continued.	**Aim**: To evaluate the 1:1 low-level community support programme ‘RIGHT4U’; to describe the characteristics of adults referred to the service; identify the impact of the service; and obtain stakeholder perceptions on the service and how it could be improved **Design**: Mixed methods (service audit and formal evaluation) **Data collection**: Online questionnaire; 1:1 interviews, focus groups **Data analysis**: Descriptive statistics; *no methodology provided for qualitative analysis* **NB**: Stakeholder consultation meetings held with 12 service users to confirm evaluation conclusions	54 adults received 1:1 support. The peer group was the most-used aspect of the service (63%). Median length of involvement 22 months for those with continued involvement and 12 months for those discharged. 27 users took part in evaluation. Overall, all participants reported at least 2 positive changes from using the service; 67% reported 4 or more, and 50% reported 8 or more. The greatest improvement was seen for increasing the amount of time spent out of the house (89%), engaging in more activities (89%), independence (78%), confidence going out in the community (74%), happiness (78%), and friendships (70%). Participants reported no change in employment or education capability. Two participants reported a single negative change (1 for friendships, and 1 for happiness) Many felt the service got them out of the house, highlighting the strength of graduated increase, and initially being accompanied by the support worker. They appreciated the new experiences and became ‘more independent’, mainly from socialising. 1:1 support was also highlighted as providing new experiences through facilitating job placements. Opportunities provided for socialising were positively regarded; participants appreciated interaction with like-minded peers and acknowledged prefering socialising with other autistic individuals who ‘understand you better’. Through others, participants reported understanding more about themselves and developing coping strategies. They reported worrying less and being able to cope with stress.	No methodology provided for qualitative data analysis; small sample; possible response bias (50% of service users represented); no participant characteristics breakdown provided for evaluation sample
Centre for Applied Autism Research (2021)	**Study type**: Grey literature report **Location**: UK **Participants**: 248 autistic adults	**Aim**: To gain a better understanding of the support needs for autistic adults and families before, during, and after the diagnostic process; and consider how these needs can be met **Design**: Mixed methods **Data collection**: Online survey with closed and open format questions (developed using literature and an expert steering group) **Data analysis**: Descriptive statistics and thematic analysis	22.1% of autistic adults surveyed were satisfied with the post-assessment phase, compared to 61.3% during assessment. 78% found this phase stressful; 75% and 61% expressed difficulties finding and understanding information on services, respectively. 30% felt they had a positive view of autism post-diagnosis. 21% had a clear understanding of next steps in terms of support. Top 5 post-diagnostic services available, accessed and found helpful: written information about autism (34.4%), support with employment or education (19.1%), contact with a charity organisation (18.3%), face-to-face support groups (14.6%), and an intervention from an autism specific support service (14.4%). Top 5 post-diagnostic support services unavailable but autistic adults wished for: intervention from an autism-specific support service (59.1%), input from mental health services (53.4%), online autism support groups (50.5%), additional support in education or employment (49.0%), and face-to-face support groups (46.9%). Six themes identified: ‘feeling alone and fighting for support’, ‘feeling overloaded with leaflets and no one to talk to’, ‘better access to ancillary services (particularly mental health)’, ‘a key person to help access services and navigate the system’, ‘autistic-led support’, and ‘better support for adults in work’. Many received little or no post-diagnostic support, reporting that diagnosis ‘makes no difference’; particularly difficult given the high levels of stress and anxiety leading up to diagnosis. Availability of post-diagnostic support services varied geographically. Many wished for access to other services, particularly for mental health, and many reported that their mental health support was lost after diagnosis. Respondents reported receiving leaflets or written information post-diagnosis, with signposting to other services, but ‘no actual help’. Many struggled navigating the system alone. Signposting was felt to be unhelpful, and even acted as a barrier. Many would prefer to have someone, like an advocate or mentor, to explain the support and help to access it, ‘to make sense of the landscape better’. The benefits of meeting and receiving support from other autistic individuals, rather than non-autistic professionals, were highlighted.	Representativeness (most respondents female, lack of ethnic diversity); self-selecting sample.
Beresford et al. (2020)	**Study type**: Journal article **Location**: UK **Participants**: 308 autistic adults; subsample of 38 interviewed	**Aim**: To describe the experiences of using an Specialist Autism Team (SAT) for individuals diagnosed and supported by a SAT compared to those who received diagnostic assessment only (DO); to describe changes in outcomes between entry into a SAT and 12 months later, explore if individual service characteristics are associated with outcomes; and explore if outcomes differ between SAT and DO group **Design**: Mixed methods **Data collection**: Questionnaires; 1:1 interviews **Data analysis**: Descriptive statistics; linear regression; ANCOVA; thematic analysis	SAT users in the diagnosis and support (D&S) and support-only (SO) cohorts received at least 1 follow-up session and a psychoeducation intervention (either 1:1 or group); the diagnosis-only (DO) cohort received 1 follow-up session but no psychoeducation. 3 SAT users didn’t receive psychoeducation due to the group environment. Many of the DO group found the follow-up session and information were inadequate, with age-inappropriate or overly negative leaflets. Signposting was described as ineffective and demoralising; all those advised to contact other services had not, noting the fact they were autistic had made this difficult. The insufficient support was particularly difficult for those shocked by the diagnosis – one described it as a ‘hit and run accident’. Individuals were positive about psychoeducation. One participant felt it ended ‘a lifetime’s worth of feeling inadequate’. Particularly positive aspects surrounded coping strategies, insight into the impact of autism and positive aspects, disclosure, and being directed to reliable sources of information. A dominant theme was the impact on how they viewed themselves: becoming more accepting, more forgiving, and feeling less need to mask behaviours; partly through identifying positive aspects. All spoke highly of psychoeducation, even after 12 months. Those in a group intervention appreciated sharing experiences and learning from others, especially those with positive stories of autism. One individual did note the prolonged wait for psychoeducation – describing this as torturous. All those who didn’t receive psychoeducation reported unresolved difficulties coming to terms with the diagnosis, and wanted further help. Some felt it negatively impacted them mentally. By contrast, those who received psychoeducation reported positive impacts of diagnosis: increased understanding of self; improved self-esteem; reduced anxiety; developing coping strategies; and reduced isolation. For the D&S group, there were small statistically significant improvements in mental health outcomes at 24 months, in managing usual activities of daily living at 24 months, and in having contact with any autism-specific third-sector organisations at 12 months. No significant improvements were found for the SO or DO group. There was some evidence of a potential worsening in mental health immediately after diagnosis for the DO group.	Unable to know which form of psychoeducation each individual received; self-selecting sample participating in interviews; underpowered sample of DO group to compare D&S and DO outcomes.
Holtom & Lloyd Jones (2019)	**Study type**: Grey literature government report **Location**: Wales **Participants**: 124 autistic adults; subsample of 43 for qualitative component	**Aim**: To evaluate the impact of the 7 Integrated Autism Services (IAS) across Wales (which provide diagnostic assessment and post-diagnostic support); to explore the experiences of autistic adults, families, and professionals accessing the IAS and services delivered **Design**: Mixed methods **Data collection**: Online survey with closed-format questions; 1:1 interviews (*n* = 5); email interviews (*n* = 5); and focus groups (*n* = 5) **Data analysis**: Descriptive statistics; *no methodology provided for qualitative analysis*	Many adults most valued the IAS being an autism-specific service, and felt relief accessing it. With the specialist nature, they felt the service was shaped around their needs. Whilst all noted the importance of a diagnosis, the response varied towards it; for some, it was appreciated that their diagnosis came from the IAS because of the post-diagnostic support provision – without which, it ‘would have been terrible’. Criticisms centred around wanting more support (rather than criticising the support they had received); specifically mentioning 1:1 mentoring and advocacy support, and access to a key-worker who could accompany them to activities, at least in the beginning. Many valued the proactive offer of support from the IAS, since in the past they had found it difficult asking for help and support. Most adults had attended at least one course, which were positively received, especially due to meeting like-minded peers who ‘also had a late diagnosis’. The amount and type of information that adults wanted varied significantly; from those who undertook significant research, to those who did not and had not read any leaflets provided. While a valuable source of information and a website are part of a solution, they were deemed insufficient if in crisis. More face-to-face or telephone contact with support workers was desired.	Participant characteristics not provided; self-selecting sample
Autism Rights Group Highlands (ARGH, 2018)	**Study type**: Grey literature report **Location**: Scotland **Participants**: 48 respondents; 41 were autistic adults	**Aim**: To explore the experiences of service users and staff of the Highland One Stop Shop (HOSS) service and its impact **Design**: Quantitative **Data collection**: Online or paper survey with closed-format questions **Data analysis**: Descriptive statistics **NB**: Qualitative data collected from free text boxes; presented as quotes but not analysed	Respondents rated each aspect of the service positively. Services rated as most impactful and beneficial were peer support (73%), social opportunities (75%), and just talking to staff (84%). Support was also received with housing, education, finances, and health. HOSS appeared to positively impact the lives of individuals. Free text responses described it as a ‘lifesaving service’. Most felt life had improved as a result (e.g., removing shame they felt about autism). Most felt their life would be negatively impacted if HOSS closed, predominantly with mental health; 3 respondents specifically mentioned suicidal ideation if the service closed. Negative comments focused only on the lack of funding and resources.	No breakdown of participant characteristics provided; small sample; limited data; descriptive statistics only (no other statistical methodology)
Crane et al. (2018)	**Study type**: Journal article **Location**: UK **Participants**: 30 adults, parents or professionals; 10 autistic adults interviewed (subsample of Jones et al., 2014 survey respondents)	**Aim**: To explore the diagnostic experiences of autistic adults in the UK; identify areas working well and areas requiring improvement; inform service-improvement recommendations **Design**: Qualitative **Data collection**: 1:1 semi-structured interviews **Data analysis**: Thematic analysis **NB**: Data for autistic adults distinguished from other interviewees	Three sub-themes were identified relating to the theme ‘inadequate post-diagnostic support provision’, ‘feeling directionless’, ‘lack of available appropriate support’, and ‘lack of emotional support’. Adults reported feeling directionless after their diagnosis, with the lack of advice and guidance being particularly difficult, and often struggling to make sense of the diagnosis and its impact. Adults experienced a general lack of support. When support was available, it was often not offered until crisis point, or felt inappropriate. When provided and felt to be useful, services were often later withdrawn. Adults reported desiring practical advice and guidance post-diagnosis, with support surrounding education, employment, housing, healthcare, and benefits. When adults were offered appropriate post-diagnostic support, it was viewed positively, though they desired more organised and overarching support, such as a ‘government-backed organisation . . . rather than variegated charities that are all struggling financially’. The combination of a general lack of support and poor signposting was often exacerbated by a lack of family support. A lack of emotional support was also an issue, particularly following the assessment process, with having gone through difficult and traumatic childhood experiences during diagnosis: ‘dredging up old stuff . . . then you’re just left to work that all out for yourself’. Support from peers who had been through the same experience was helpful.	Representativeness
Hickey et al. (2018)	**Study type**: Journal article **Location**: UK **Participants**: 13 autistic adults	**Aim**: To explore the experiences of autism in later adulthood (aged over 50) **Design**: Qualitative **Data collection**: 1:1 semi-structured interviews **Data analysis**: Thematic analysis	Three themes identified, with one sub-theme relating to the post-diagnosis period: ‘reaching out (post-diagnosis)’. Post-diagnosis, most participants had some involvement in autism groups or connection with other autistic individuals. For most, this was in a deliberate effort to gather additional information about autism and how it applied to their lives. For some, reaching out was an attempt to increase their social engagement. Furthermore, meeting other autistic adults offered a sense of shared experience and understanding, which was missing in any existing social networks. The importance of acceptance and universality of experience was mentioned by most participants: ‘you’re accepted’ and felt they did not have to hide, as they were ‘in the same boat’. Attending autism groups allowed certain experiences to be normalised, removing shame.	Representativeness; recruited from local autism support/social groups so possible selection bias.
Southby & Robinson (2018)	**Study type**: Journal article **Location**: England **Participants**: 14 autistic adults **Service evaluated**: Leeds AIM is a third-sector, locally-commissioned, organisation. Co-led by autistic adults, it serves as an ‘autism hub’ – providing information, signposting and advocacy services; alongside facilitating training sessions, employment support, and peer-support groups, amongst other services.	**Aim**: To consider the efficacy of providing low-level support in the form of information and signposting, advocacy, and mentoring to adults with ‘high-functioning’ autism spectrum disorder **Design**: Qualitative **Data collection**: 1:1 semi-structured interviews **Data analysis**: Thematic analysis **NB**: Intended to be a mixed methods evaluation, but sample size small and decision made to not analyse quantitative data	Five themes identified: ‘employability, education and volunteering’, ‘access to information and support’, ‘reduced social isolation’, ‘health and wellbeing’, and ‘managing day-to-day’. Participants reported a significant outcome was improved access to information and support, with easy accessibility and empowerment to take appropriate action. Positive impacts on employability and access to education, particularly through an employment peer-support group, social skills training, and CV building, alongside facilitated volunteer and work experience opportunities. A positive impact was found on socialisation. Participants found it allowed them to interact with ‘like-minded people’, finding it easier to ‘get on with other autistic people’ compared to non-autistic people. Friendships were gained through the service, and outside of it, via improved confidence and communication skills. Participants noted improved health and wellbeing with improved confidence and reduced anxiety related to service use. One user indicated that the service’s support prevented them from self-harming and attempting suicide. The service also advocated for users’ access to healthcare. Overall, participants felt it aided management of everyday life, instead of struggling; enabling them to live ‘less-chaotic lives’ through development of strategies, particularly through peer mentoring. It allowed them to be more tolerant of themselves and appreciate their positive aspects.	Representativeness; small sample; data collected from only one service; data collected at one point in time
Bracher (2014)	**Study type**: Unpublished research project **Location**: England **Participants**: 11 autistic adults	**Aim**: To evaluate the experiences and needs of autistic adults for diagnostic assessment and post-diagnosis; and to highlight areas for service improvement, particularly regarding post-diagnostic support needs **Design**: Mixed methods **Data collection**: Questionnaire with two instruments (four ONS wellbeing questions, and the WHO Quality of Life Brief survey); 1:1 semi-structured interviews **Data analysis**: Descriptive statistics; interpretative analysis	Participants had lower-than-average levels of wellbeing and quality of life, with particularly low levels of psychological quality of life. Only one feedback session and a diagnostic report with recommendations were provided. Most expressed a degree of satisfaction with the report, but levels of engagement with it were highly variable. A distinction was made by participants between the experiences of the feedback session and the report. The feedback session expressed autism positively, whilst the report was thought to be framed negatively. For all participants, the most important outcome of diagnosis was obtaining appropriate post-diagnostic support. However, for most, this was not received. Participants felt unable to identify and access services, and did not know ‘which way to turn’ following diagnosis. Several felt confused about what support they needed; whilst they could identify a general problem, they were unclear about the practical steps necessary to deal with these issues. Participants felt the signposting information provided by the service needed to be improved and to be more comprehensive. For those who had found support from other autistic individuals (e.g., though support groups), this was positively regarded.	Small sample size; non–peer reviewed; largely male, unrepresentative sample; other participant characteristics not provided
Jones et al. (2014)	**Study type**: Journal article **Location**: UK **Participants**: 128 autistic adults	**Aim**: To explore the experiences of autistic adults diagnosed with autism in the UK, including post-diagnostic support received and desired **Design**: Quantitative **Data collection**: Online survey with closed-format questions and two instruments (Beck Depression Inventory, and Beck Anxiety Inventory) **Data analysis**: Descriptive statistics and multiple regression analysis **NB**: Qualitative data collected from free text boxes and presented as quotes, but not formally analysed	A large minority received no post-diagnostic support at all (41.9%). For those offered support, most commonly offered types were: support groups (21.9%), counselling (21.7%), financial advice (13%), support at school or work (12.4%), social skills training (7.8%), community care assessment (3.1%), and housing advice (1.6%). This contrasted with the percentage of individuals who wanted access to such services: counselling (44.5%), social skills training (36.7%), support groups (35.9%), support at school or work (34.4%), financial advice (29.7%), input from healthcare professionals (22.7%), community care assessment (20.3%), and housing advice (16.4%). Thus, there was a large degree of unmet need. 46.9% of autistic adults were satisfied with the diagnostic process. Satisfaction with support was the area of least satisfaction (22.6% satisfied); this was significantly associated with overall satisfaction on multiple regression analysis.	Self-selecting sample of respondents; retrospective; possible response bias (more likely to respond if polarising experiences); no way of knowing characteristics or views of non-respondents; largely promoted through support services

SST: social skills training; NHS: National Health Service; SAT: Specialist Autism Team; ANCOVA: analysis of co-variance; ARGH: Autism Rights Group Highlands.

a Studies may have included other participants. Only data related to adult participants are included here.

b Studies may have reported other findings. Only findings related to post-diagnostic support are included here.

### Quality assessment

Overall, the quality of journal articles was reasonable (see Supplemental Material C). Qualitative studies tended to be of higher quality. Issues within quantitative and mixed methods studies included the sample being unrepresentative of the autistic adult population (*n* = 4) and the risk of non-response bias (*n* = 4). Participants in the qualitative studies did not represent the entire autistic adult population, but qualitative findings are not intended to be generalisable. Grey literature study quality was mixed and generally lower (see Supplemental Material D); limitations included the absence of references (*n* = 2) or stated methodology (*n* = 1).

### Participant demographic characteristics

In total, 1,607 autistic adults were represented across studies. The mean participant age was 42.68 years (range = 17–89 years), and 51.01% of participants identified as male. Participants were predominantly of White ethnicity (92.78%).

### Efficacy of post-diagnostic support

Six studies evaluated two types of post-diagnostic support: low-level support services (McConkey et al., 2021; Southby & Robinson, 2018) and psychoeducation (Beresford et al., 2020; Crane et al., 2023; Crane et al., 2021; Hatton & Lee, 2022). However, none collected data for defined outcomes to enable assessment of the efficacy of such post-diagnostic support. As such, we cannot comment on the efficacy of any of the support options identified here.

### Experiences of post-diagnostic support

#### General experiences

Adults’ experiences were often negative, with many reporting a lack of access to post-diagnostic support (Crowson et al., 2024). In a survey by Jones et al. (2014), 41.9% of the 128 respondents received no post-diagnostic support. When support was offered, it failed to correlate with what they desired (Jones et al., 2014). In another survey involving 343 autistic adults producing consensus statements on their experience and preferences of post-diagnostic support, most did not receive any ‘optimal’ support as defined by key stakeholders (Wigham et al., 2022; see Table 1 for more detail). This mirrored a large study by the Westminster Commission, with a misalignment between support available and desired; only 22.1% expressed satisfaction with the post-diagnostic phase (Centre for Applied Autism Research, 2021). Autistic adults in the study conducted by Hull et al. (2024) felt that the support they were offered was inflexible and needed greater personalisation, sometimes being inappropriate for certain individual’s needs.

A recent study by Crowson et al. (2024) used a modified Delphi method to generate a consensus from the autistic community on their post-diagnostic support priorities. Twenty-four key priorities were identified, which represented important elements that autistic adults wished to be offered in the context of post-diagnostic support. In particular, participants prioritised being able to access any support in their area in the first place, followed by feeling assured that professionals involved in offering services were adequately trained. Third, participants wanted to receive support with processing the impact of receiving an autism diagnosis in adulthood, followed by experiencing consistency in use of their preferred methods of contact, and being provided with an individualised support plan.

Adults did describe positive experiences and reported tangible benefits when appropriate post-diagnostic support was received (Crane et al., 2018, 2021, 2023). A recent Scottish evaluation of their national post-diagnostic support service, through which everyone can access support, sought the opinions of autistic adults via an online survey (Minister for Mental Wellbeing and Social Care, 2022). While it is unknown which specific support each received, impacts were largely positive: most commonly providing a better understanding of autism (90%) and themselves (87%), and in forming connections with autistic peers (67%). In addition, in an evaluation of the seven Welsh Integrated Autism Services, adults specifically praised services for their proactive offer of support (Holtom & Lloyd Jones, 2019).

#### Follow-up and signposting

Despite being the most common form of support provided, the sole provision of a follow-up appointment with signposting to other services appeared unfavourable. In a large evaluation, the SHAPE project (Supporting adults with High-functioning Autism and asPerger syndromE), participants either received post-diagnostic psychoeducation, or a diagnosis only; the diagnosis-only group received one follow-up session, leaflets, and signposting (Beresford et al., 2020). Many found the information inappropriate, and simple signposting was also deemed demoralising; none had contacted the recommended services, noting that being autistic made this challenging. One adult described the diagnostic process as ‘invasive’, and compared the subsequent lack of post-diagnostic support to a ‘hit and run accident’ (p. 83; Beresford et al., 2020).

Similarly, in a small grey literature evaluation of an NHS diagnostic service, participants desired more comprehensive signposting (Bracher, 2014). They were often able to identify a general problem, but were unsure of the necessary steps to manage such issues without the support of others (Bracher, 2014). Westminster Commission survey respondents likewise frequently received leaflets or signposting, but indicated that this afforded ‘no actual help’ (p. 52; Centre for Applied Autism Research, 2021).

#### Low-level support

Low-level support services take the provision of information and signposting further by allowing autistic individuals to utilise resources that are appropriate to them, with the support and advocacy of others, either on a 1:1 basis or as part of a larger service.

Two studies evaluated such services (described in Table 1). Southby and Robinson (2018) evaluated ‘Leeds AIM’, an autism hub, and McConkey et al. (2021) evaluated ‘RIGHT4U’, a 1:1 service. A significant outcome reported by Leeds AIM users was improved access to appropriate information and empowerment to act upon this, reporting positive impacts on access to education and employment. While both services facilitated access to opportunities (e.g., work experience), RIGHT4U users disclosed no changes to their employment or education status. This may be due to greater opportunities within Leeds AIM (e.g., through an employment peer-support group). Positive outcomes of service use identified by participants in both studies included reduced anxiety, improved confidence, increased social opportunities, development of coping strategies, and improved access to healthcare.

The ‘autism hub’ setup is seen within Scotland, with eight ‘One Stop Shops’ (integrated information hubs and advice services, offering information, guidance, access to other services, and training opportunities for autistic people and their families). In a small survey evaluating the Highland One Stop Shop, autistic adults rated the service highly, with positive results for peer support, socialising, and staff ratings (ARGH, 2018). Many felt it improved their mental health, including addressing their previously unsupported suicidal ideation (ARGH, 2018). One Leeds AIM user also stated the support prevented them from self-harming and attempting suicide (Southby & Robinson, 2018).

#### Psychoeducation

Five studies evaluated psychoeducation as post-diagnostic support (described in Table 1). Hatton and Lee (2022) qualitatively evaluated an NHS-delivered 7-week online group psychoeducation programme. Participants described an opportunity for knowledge sharing, consolidation, and self-reflection, subsequently allowing them to ‘manage rather than struggle’ (Hatton & Lee, 2022). Improved daily management was reflected in the evaluation by Crane et al. (2021) of an autistic-led 10-week group psychoeducation programme (*‘Exploring Being Autistic*’; see Table 1 for more details). The programme was described by one participant as enabling them to ‘see what’s happening and find a way out’ of problematic situations (p. 900).

Within the SHAPE study, autistic adults receiving psychoeducation recounted positive experiences (Beresford et al., 2020). A dominant theme was the impact on their self-perception; becoming more accepting and enhancing their self-esteem. Echoing Hatton and Lee (2022) and Crane et al. (2021), other significant benefits centred around developing coping strategies and learning from others. Participants of all three studies reported acquiring a sense of community, reduced isolation, and less need to mask (masking being the concealing of autistic traits or difficulties, which is often emotionally and physically taxing (Cook et al., 2021)).

In contrast, the subgroup of participants in the SHAPE study who did not receive psychoeducation reported unresolved difficulties post-diagnostically, particularly in accepting their diagnosis (Beresford et al., 2020). This negatively impacted the mental health of some, which they attributed to this lack of psychoeducation. From quantitative data, there was some evidence of deteriorating mental health immediately after diagnosis for this cohort, compared to a small but statistically significant improvement in mental health outcomes for those receiving psychoeducation (Beresford et al., 2020).

The autistic-led nature of the psychoeducation programme ‘*Exploring Being Autistic*’ was highlighted as a strength, in both the in-person and online versions of the programme (Crane et al., 2023; Crane et al., 2021). The autistic facilitator was deemed more understanding, and provided a positive role model for recently diagnosed participants. SHAPE study participants also appreciated hearing positive stories of autism, while participants in the study reported by Hatton and Lee (2022) felt the content should be more positive in their non-autistic-led programme (Beresford et al., 2020). In replicating their study of the *Exploring Being Autistic* programme when delivered online as opposed to in-person, Crane et al. (2023) noted some unique benefits of online delivery, including increased potential accessibility, reduced cognitive load/preservation of participants’ energy for focusing on the programme, and the opportunity to form meaningful social connections with other group members. However, participants also emphasised the importance of flexibility in delivery format, as technological issues had the potential to hinder access, and some group-based activities (e.g., the use of breakout rooms) were felt to be less effective online (Crane et al., 2023).

Hatton and Lee (2022) explored participants’ preferences for psychoeducation delivery. Some felt online delivery was practical and accessible, and lessened anxiety. Others, however, considered that it did not permit sufficient scope for interaction.

#### Peer support

No studies directly evaluated peer-support programmes. However, peer support was a theme common to most studies, with support groups being desired by autistic people, and with significant impacts arising from the support of like-minded individuals (Centre for Applied Autism Research, 2021; Jones et al., 2014).

For example, Crompton et al. (2022) explored post-diagnostic support in interviews with autistic adults. Many thought that peer support would lessen the difficulties of processing the diagnosis by validating their experiences (Crompton et al., 2022). Similarly, in the study by Hickey et al. (2018), a common thread in the experiences of older autistic adults (aged 50+) was the importance of universality; being ‘in the same boat’. In an exploration of the diagnostic experiences of autistic adults by Crane et al. (2018), the perceived invasive nature of the diagnostic process (‘dredging up old stuff’) was described as traumatic (p. 3768), and subsequent peer support found to be beneficial.

Within several studies, autistic people reported more comfortable relationships with other autistic individuals, with a lessened feeling of the need to mask, thereby improving self-perceived confidence and communication skills (Cook et al., 2021; Crane et al., 2021; Hatton & Lee, 2022). In addition, knowledge sharing helped to formulate coping strategies, with some considering sharing with peers to be more critical than the structured educational components of psychoeducation (Crane et al., 2021). Peer-led support was also seen as more authentic by participants in the study by Hull et al. (2024), resulting in a more positive experience of engaging with support overall.

As with psychoeducation, the preference was for autistic-led peer support (Crompton et al., 2022; Hull et al., 2024). However, individuals acknowledged that being autistic does not inherently provide the knowledge or skills necessary for the role, and that this requires training and/or experience. Limitations were likewise noted, particularly in peer-support not being used to replace specialist support, such as from mental health services (Crompton et al., 2022).

### Service mapping

**Figure 3.**
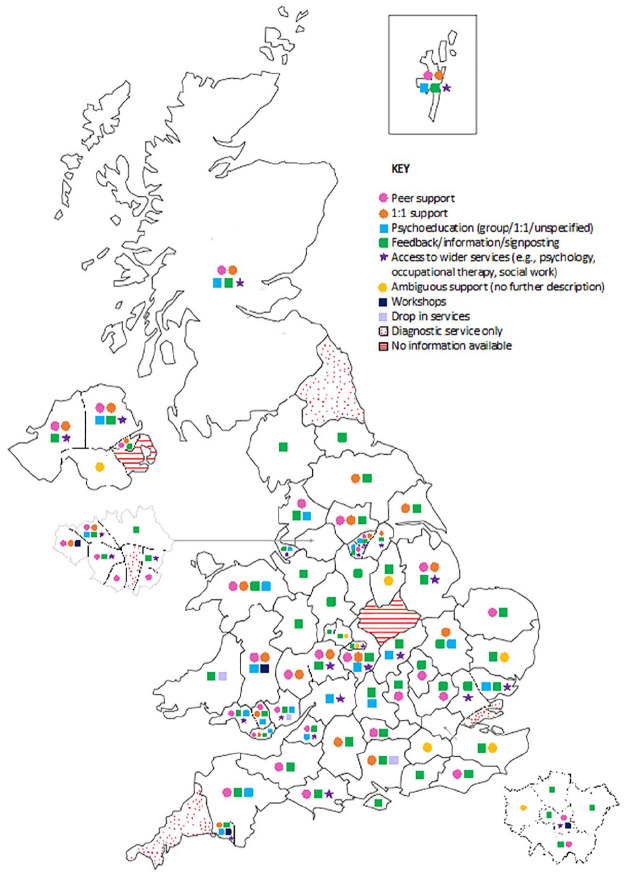
Map of the UK, with each symbol denoting the type of post-diagnostic support available in each region.

Systematic online searches identified 83 distinct services for autistic adults without intellectual disabilities. These included services offering diagnosis only (*n* = 6), diagnosis and support (*n* = 71), and support only (*n* = 6). Services covered all regions of the UK except two: County Down in Northern Ireland, and Leicestershire in England, where no information was found. The type and extent of post-diagnostic support varied geographically; this is visually represented in Figure 3.

Of these 83 services, 93% reported providing post-diagnostic support. Most support concerned feedback, information or signposting (83%); some offered this exclusively (17%). Many provided either peer or 1:1 support (58%); most commonly peer-group support (49%). Some offered psychoeducation (31%), usually group psychoeducation (23%). One-third reported access to wider services, including psychology, occupational therapy, speech and language therapy, or social workers (34%). Few provided all of these forms of support (9%). Infrequently, drop-in services and workshops were also provided (9%).

## Discussion

This study explored the content, provision, and evidence for post-diagnostic support for autistic adults receiving a diagnosis in adulthood in the UK, using a combination of systematic review of studies, and service mapping of available support.

### Availability of support

Post-diagnostic support is available across most of the UK. However, the extent of support is variable, with some regions providing none or only a minimal amount (e.g., one post-diagnosis follow-up appointment to signpost to other services). Despite apparent provision, autistic adults frequently describe difficulties receiving support, indicating that availability does not necessarily translate to accessibility. UK healthcare professionals have previously highlighted that they are too overstretched and underfunded to provide adequate support, and that even when this is available, they are often pressured not to offer it (Crane et al., 2018; Rogers et al., 2016). This reflects findings internationally; in a recent European Union survey, most professionals reported the availability of post-diagnostic support, yet most autistic adults had received none (Scattoni et al., 2021).

### Types of support

Follow-up and signposting represent the most common forms of post-diagnostic support. Although recommended by the National Institute for Health and Care Excellence (NICE), this alone appears insufficient and unacceptable to autistic people (National Institute for Health and Care Excellence, 2021). Autistic adults report difficulties reaching out to signposted services due to the very characteristics of autism; indeed, such help-seeking difficulties are likewise described as barriers to accessing mental health support (Camm-Crosbie et al., 2019).

Instead, autistic adults wish for a low-level support service, where they can access appropriate information and services accompanied by the advocacy of others. Low-level support from ‘autism hubs’ comprises the approach recommended in the previous Adult Autism Strategy (2014), and appears to improve multiple dimensions of autistic adults’ lives (Department of Health and Social Care et al., 2014). Indeed, where it is not accessed, low-level support is frequently desired (Centre for Applied Autism Research, 2021; Crane et al., 2018; Holtom & Lloyd Jones, 2019).

In addition, psychoeducation appears to have the potential to enhance self-esteem and self-acceptance, allowing adults to come to terms with their diagnosis, develop coping strategies, and improve the management of their day-to-day lives. Psychoeducation has been effective for other conditions, such as ADHD, schizophrenia, and bipolar disorders (Buizza et al., 2019; Hirvikoski et al., 2017; Xia et al., 2011). However, a manualised psychoeducation programme for autism requires development. Psychoeducation could also be integrated into other types of post-diagnostic support offered, for instance, mental health support. Up to 40% of autistic adults experience co-occurring mental health conditions (Lai et al., 2019), and may receive support through traditional mental health services. In addition to being aware of how autism may impact individuals’ experiences of mental health difficulties and potential treatment, ongoing mental health support may provide a practical context through which to provide post-diagnostic support. For instance, autistic adults interviewed about social skills training (although not necessarily in a post-diagnostic context) recommended that this type of support be integrated within mental and physical health support to meet needs in a holistic way (Hull et al., 2024). However, we note that mental health support for autistic adults also comes with limitations, including a lack of evidence, lack of provision, and long waiting times (Linden et al., 2023).

While not directly evaluated, peer support emerges as a critical aspect of other post-diagnostic support methods to autistic adults. Many desired such groups in order to build social connections and learn through the experiences of others (Crompton et al., 2022; Hickey et al., 2018).

### Autistic-led support

Among the studies in this review, there was a call for autistic-led post-diagnostic support; to provide better understanding, highlight the strengths of being autistic, and to offer positive role models. A desire for this positive framing of autism in post-diagnostic support was reiterated throughout many studies (ARGH, 2018; Campbell et al., 2020; Cook et al., 2021; Crane et al., 2021, 2023; Hatton & Lee, 2022; Holtom & Lloyd Jones, 2019; McConkey et al., 2021). Notably, autistic adults consistently reported greater ease in communicating with other autistic peers. While communication difficulties define an autism diagnosis, recent evidence has shown that peer-to-peer information transfer among autistic people can be highly efficient, suggesting that post-diagnostic support co-produced and led by autistic people may provide benefits (Crompton et al., 2020; Hull et al., 2024).

### Strengths and limitations

Strengths of this systematic review included the use of a comprehensive and sensitive search strategy in multiple databases and the inclusion of grey literature, which is commonly absent from reviews and can add valuable information. Furthermore, duplicate screening added rigour. In addition, the included studies were predominantly published within the last 7 years, providing up-to-date evidence. Finally, using a narrative synthesis allowed the integration of all available evidence when meta-analysis proved inappropriate.

However, limitations exist. Primarily, the lack of evaluations of post-diagnostic support is notable, although this also highlights its nature as an emerging research area. Existing evaluations are mostly small-scale and lack defined outcomes accompanied by efficacy data; as a result, this review was not able to evaluate the efficacy of post-diagnostic support. The variable quality of studies is also noteworthy; while the inclusion of grey literature is beneficial, the research being referred to was often of lower quality. In addition, several studies relied on self-selecting samples of autistic people to provide qualitative data on experiences of post-diagnostic support, which may impact upon study conclusions and recommendations. Moreover, despite the efforts made, the identification of support availability depended on services publishing such information and keeping this up-to-date, and therefore could be inaccurate. Finally, there is a notable underrepresentation of non-White ethnicities within the sample of participants, limiting the generalisability of findings to other ethnic groups (Hirvikoski et al., 2017).

### Implications

#### Commissioning support and its development

Given the potential impact of not receiving post-diagnostic support, commissioning sufficient post-diagnostic support is essential. While requiring initial investment, the subsequent reduced demand for wider services (e.g., mental health) may balance this cost by preventing adults from reaching crisis point. However, a critical first step is funding the full evaluation of current support provisions and expanding or refining existing support as needed. The impact of the funding promised in the latest Autism Strategy will require investigation (Department of Health and Social Care and Department for Education, 2021).

Importantly, this review did not aim to identify the ‘best’ method of post-diagnostic support; those discussed should not be considered mutually exclusive. The post-diagnostic needs of autistic adults may evolve and depend on multiple factors (e.g., availability of family support, or the presence of co-occurring conditions), which may account for differing forms of post-diagnostic support desired by adults within this review. Thus, each may require something different from a post-diagnostic service; a spectrum of long-lasting support is required, rather than time-limited interventions.

#### Individual choice

Where explored, preferences varied for online versus face-to-face post-diagnostic support. Online support may improve accessibility and diminish anxiety, but could limit social interaction, and reliable internet access is also essential to consider (Baweja et al., 2022; Hatton & Lee, 2022). For group versus individual support, 11 consensus statements created by autistic adults and professionals recommend that post-diagnostic support be available in both formats, allowing for individual choice (Wigham et al., 2022).

#### Organisation

The provision of post-diagnostic support varies, creating a ‘postcode lottery’ in terms of access. Two UK countries currently have overarching, government-funded post-diagnostic support services: Scotland, with a national pilot service provided by third-sector organisations; and Wales, with seven regional services. While we cannot draw definite conclusions from these evaluations, a centrally-organised system could reduce inequality of access. Furthermore, many third-sector organisations are autistic-led or co-led, and commissioning such services nationwide could contribute to the creation of an expert post-diagnostic service.

### Future research

Limitations of this review include the lack of efficacy evaluations and the absence of representative populations, predominantly of non-White ethnicities; the latter, unfortunately, has been the norm within autism research (Steinbrenner et al., 2022). High-quality evaluations with comparable outcomes are required to provide appropriate evidence for the efficacy of support currently offered, and to determine the impact for those not represented by current studies.

In addition, research should consider comparing post-diagnostic service provision across different countries. The present study focused on the UK due to its unique national healthcare system, and a limited capacity to conduct an international systematic review. Anecdotally, the majority of research in this area seems to be based in the UK. However, there are other locations where post-diagnostic support for adults has been evaluated, including across the European Union (e.g., Scattoni et al., 2021). Future research should expand comparisons beyond a single nation in order to synthesise the best evidence for post-diagnostic support for autistic adults.

## Conclusions

This review is the first to appraise the evidence surrounding post-diagnostic support for autistic adults without intellectual disabilities diagnosed in adulthood in the UK. It highlights the need for adequate post-diagnostic support, and the positive impact this may have on the lives of autistic adults. Low-level support, psychoeducation, and peer-support methods appear acceptable and feasible; and when experienced, autistic adults appreciate these services. However, few post-diagnostic programme evaluations exist; thus, the ability to draw firm conclusions about their efficacy is limited. Nevertheless, our findings contribute to an emerging area of research. Further research is needed to develop manualised post-diagnostic support programmes and assess their efficacy. Such research should involve representative populations, to meet the needs of all autistic adults.

## Supplemental Material

sj-docx-1-aut-10.1177_13623613241273073 – Supplemental material for Post-diagnostic support for adults diagnosed with autism in adulthood in the UK: A systematic review with narrative synthesis Supplemental material, sj-docx-1-aut-10.1177_13623613241273073 for Post-diagnostic support for adults diagnosed with autism in adulthood in the UK: A systematic review with narrative synthesis by Jade Eloise Norris, Rebecca Harvey and Laura Hull in Autism

## Notes

1. Intellectual disability is defined by the National Institute for Health and Care Excellence (2015) and Department of Health and Social Care (2001) as a significantly reduced ability to understand new or complex information and learn new skills (usually with an IQ of <70), with a reduced ability to cope independently, and onset in childhood.
