# Supplementary material for: Post-diagnostic support for adults diagnosed with autism in adulthood in the UK: A systematic review with narrative synthesis
Source: Autism. 2024 Sep 10;29(2):284–309. doi: 10.1177/13623613241273073 (PMC11816465; doi:10.1177/13623613241273073)
Supplement: sj-docx-1-aut-10.1177_13623613241273073 – Supplemental material for Post-diagnostic support for adults diagnosed with autism in adulthood in the UK: A systematic review with narrative synthesis [file sj-docx-1-aut-10.1177_13623613241273073.docx]

**Supplemental Materials A: Biomedical database search strategies.**

|  | Ovid MEDLINE(R) 1946-present;  APA PsycINFO 1806-2024 | | CINAHL | | Web of Science | |
| --- | --- | --- | --- | --- | --- | --- |
| Condition | 1 | autis*.mp or “autis* spectrum disorder”.mp or “autis* spectrum condition”.mp or “autis* disorder”.mp or “ASD”.mp or “ASC.”mp or “asperger*”.mp or “atypical autism”.mp or “pervasive development* disord*”.mp or “PDD-NOS”.mp or exp Autistic Disorder/ or exp Autism Spectrum Disorder/ or exp Asperger Syndrome/ | 1 | autis* or "autis* spectrum disorder" or "autis* spectrum condition" or "autis* disorder" or "atypical autism" or ASD or ASC or "asperger*" or "asperger syndrome" or "asperger disorder" or “pervasive development* disorder*” or "PDD-NOS" | 1 | ((((((((ALL=(autis*)) or ALL=(autism spectrum condition)) or ALL=(autism spectrum disorder)) or ALL=(ASD)) or ALL=(ASC)) or ALL=(autistic disorder)) or ALL=(asperger*)) or ALL=(atypical autism)) or ALL=(PDD-NOS)) or ALL=(pervasive development* disorder*) |
| Population | 2 | adult*.mp or “over 18”.mp or “diagnos* in adult*”.mp or exp Adult/ | 2 | adult* or “over 18” | 2 | ((ALL=(adult) or ALL=(over 18))) |
| Intervention | 3 | diagnos*.mp or post-diagnos*.mp or “diagnos* service*’.mp or model*.mp or support*.mp or service*.mp or intervention*.mp or therap*.mp or group*.mp or counsel*.mp or toolkit*.mp or train*.mp or approach*.mp or “social learning”.mp or “peer led”.mp or “autis* led”.mp or “peer support”.mp or activit*.mp or communicat*.mp or online.mp or psychosocial*.mp or psychoeducat*.mp or social*.mp or pathway*.mp or “specialist service”.mp or (integrat* adj3 car*).mp or (collaborat* adj3 car*).mp or (shar* adj3 car*).mp 1 | 3 | (diagnos* or "post-diagnos*" or model* or support or service* or intervention* or therap* or group* or counsel* or online or toolkit* or train* or approach* or "social learning" or "peer led" or "autis* led" or activit* or communicat* or psychosocial* or psychoeducat* or social* or pathway* or "specialist service*" or (integrat* N3 car*) or (collaborat* N3 car*) or (shar* N3 car*)) | 3 | (((((((((((((((((((((((((ALL=(diagnos*)) OR ALL=(post-diagnos*)) OR ALL=(model*)) OR ALL=(support*)) OR ALL=(service*)) OR ALL=(intervention*)) AND ALL=(online)) OR ALL=(communicat*)) OR ALL=(therap*)) OR ALL=(group*)) OR ALL=(counsel*)) OR ALL=(toolkit*)) OR ALL=(train*)) OR ALL=(approach*)) AND ALL=(activit*)) OR ALL=("social learning")) OR ALL=("peer led")) OR ALL=("autis* led")) OR ALL=(psychosocial*)) OR ALL=(psychoeducat*)) OR ALL=(social*)) OR ALL=(pathway*)) OR ALL=("specialist service")) OR ALL=((integrat* "near" care))) OR ALL=((collaborat* "near" care))) OR ALL=((shared "near" care)) |
| Combination | 4 | (1 and 2 and 3) not “adult spinal deformity” | 4 | 1 and 2 and 3 | 4 | 1 and 2 and 3 |
| Limit | 5 | Limit to (English language and yr=”2012-Current”) | 5 | Limit to published 2012-2024 | 5 | Limit Publication Years to 2012-2024 |

**Supplemental Materials B: Grey literature search strategies.**

|  | Google Scholar | EThOS | ProQuest |
| --- | --- | --- | --- |
| Search | With all words anywhere in the article: autism adult post diagnostic support | All fields: autism and adult and post diagnostic | Anywhere: autism and adult and (post diagnostic) |
| Limit | Published 2012-2024 | 2012-2024 | Publication date last 10 years, English language, University/institution location UK |

**Supplemental Materials C: Quality assessment results for journal articles using MMAT.^16^**

|  | Screening | | Qualitative | | | | | Quantitative descriptive | | | | | Mixed methods | | | | |
| --- | --- | --- | --- | --- | --- | --- | --- | --- | --- | --- | --- | --- | --- | --- | --- | --- | --- |
|  | **Clear research questions?^a^** | **Do the collected data allow the research question to be addressed?** | **Appropriate approach?** | **Data collection methods adequate?** | **Findings adequately derived from the data?** | **Interpretation of results substantiated by data?** | **Coherence between data sources, collection, analysis, interpretation?** | **Relevant sampling strategy?** | **Sample representative of target population?** | **Measurements appropriate?** | **Risk of non-response bias?** | **Statistical analysis appropriate to answer research questions?** | **Adequate rationale for mixed methods?** | **Components effectively integrated?** | **Outcomes of integrated components adequately interpreted?** | **Divergences and inconsistencies addressed?** | **Adhering to the criteria of each method?** |
| Hull (2024) |  |  |  |  |  |  |  |  |  |  |  |  |  |  |  |  |  |
| Crowson (2024) |  |  |  |  |  |  |  |  |  |  |  |  |  |  |  |  |  |
| Crane (2023) |  |  |  |  |  |  |  |  |  |  |  |  |  |  |  |  |  |
| Crompton (2022) |  |  |  |  |  |  |  | ·· | ·· | ·· | ·· | ·· | ·· | ·· | ·· | ·· | ·· |
| Wigham (2022) |  |  | ·· | ·· | ·· | ·· | ·· |  |  |  |  |  | ·· | ·· | ·· | ·· | ·· |
| Crane (2021) |  |  |  |  |  |  |  | ·· | ·· | ·· | ·· | ·· | ·· | ·· | ·· | ·· | ·· |
| Hatton (2021) |  |  |  |  |  |  |  | ·· | ·· | ·· | ·· | ·· | ·· | ·· | ·· | ·· | ·· |
| McConkey (2021) |  |  |  |  |  |  |  |  |  |  |  |  |  |  |  |  |  |
| Beresford (2020) |  |  |  |  |  |  |  |  |  |  |  |  |  |  |  |  |  |
| Crane (2018) |  |  |  |  |  |  |  | ·· | ·· | ·· | ·· | ·· | ·· | ·· | ·· | ·· | ·· |
| Hickey (2018) |  |  |  |  |  |  |  | ·· | ·· | ·· | ·· | ·· | ·· | ·· | ·· | ·· | ·· |
| Southby (2018) |  |  |  |  |  |  |  | ·· | ·· | ·· | ·· | ·· | ·· | ·· | ·· | ·· | ·· |
| Jones (2014) |  |  | ·· | ·· | ·· | ·· | ·· |  |  |  |  |  | ·· | ·· | ·· | ·· | ·· |

Code: green = criteria met; orange = cannot tell; red = criteria not met; ·· = not applicable (criteria not relevant).

^a^Note: largely due to qualitative or exploratory nature of included studies, no study specifically stated research questions; all were reported as aims/objectives. For the purposes of this review, this was interpreted as meeting this criterion.

**Supplemental Materials D: Quality assessment results for grey literature using AACODS.^17^**

|  | Authority | | Accuracy | | | | | | | | | | Coverage | Objectivity | | Date | | | Significance | | | | | | |
| --- | --- | --- | --- | --- | --- | --- | --- | --- | --- | --- | --- | --- | --- | --- | --- | --- | --- | --- | --- | --- | --- | --- | --- | --- | --- |
|  | **Reputable?** | **Reference list?** | **Aim/brief?** | **Aim/brief met?** | **Stated methodology?** | **Methodology adhered to?** | **Peer-reviewed?** | **Edited by reputable authority?** | **Credible sources and reference?** | **Representative of work in field?** | **Data collection explicit?** | **Unbiased interpretation?** | **Any limits stated?** | **Bias of author?** | **Balanced presentation?** | **Date stated?** | **Valid reason for absence?** | **Contemporary material?** | **Meaningful?** | **Add context?** | **Add something unique?** | **Strengthen or refute current position?** | **Research area lesser without it?** | **Integral, representative, typical?** | **Impact?** |
| Beresford (2023) |  |  |  |  |  |  |  |  |  |  |  |  |  |  |  |  |  |  |  |  |  |  |  |  |  |
| Scottish Government (2022) |  |  |  |  |  |  |  |  |  |  |  |  |  |  |  |  |  |  |  |  |  |  |  |  |  |
| Westminster Commission (2021) |  |  |  |  |  |  |  |  |  |  |  |  |  |  |  |  |  |  |  |  |  |  |  |  |  |
| Holtom (2019) |  |  |  |  |  |  |  |  |  |  |  |  |  |  |  |  |  |  |  |  |  |  |  |  |  |
| ARGH (2018) |  |  |  |  |  |  |  |  |  |  |  |  |  |  |  |  |  |  |  |  |  |  |  |  |  |
| Bracher (2014) |  |  |  |  |  |  |  |  |  |  |  |  |  |  |  |  |  |  |  |  |  |  |  |  |  |

Code: green = criteria met; orange = cannot tell; red = criteria not met.

**Supplemental Materials D: Aggregated participant characteristics**

|  | Gender^a,b^ | Age | Diagnosed in adulthood | Age at diagnosis | Ethnicity |
| --- | --- | --- | --- | --- | --- |
| Mean | M: 51.01%  F: 44.98%  NB/other/did not say: 8.05% | 41.68 years | 90.85% | 36.65 | 92.78% White (British/Irish/other) |
| Number of studies contributing | 16 | 13 | 12 | 5 | 9 |

^a^F = female; M = male; NB = non-binary; ^b^ Manuscript reported percentages

**Supplemental Materials F: Breakdown of participant characteristics**

| Author (year) | Sample size | Gender | Age (years) | Diagnosis details | Ethnicity | Sampling/recruitment |
| --- | --- | --- | --- | --- | --- | --- |
| Hull (2024) | 11 | 45.45% male; 45.45% female; 9.09% agender | Mean 37.50 Range 20-58 | 27.27% diagnosed in childhood/adolescence; 72.72% diagnosed in adulthood | 90.91% White British/Scottish/ European/American  9.09% no response | Recruited via purposive sampling using social media, local autism support  services, and snowball sampling |
| Crowson (2024) | *Round 1:* 43  *Round 4:* 113 | *Round 1:*  56.00% male;  26.00% female;  18.00% non-binary/other/prefer not to say;  *Round 4:*  31.00% male;  61.00% female;  8.00% non-binary/other | *Round 1*:  Mean 38.71  Range 19-60  *Round 4:*  Mean 36.60  Range 19-67 | All diagnosed in adulthood, and within the last 10 years | *Round 1:*  77.00% White;  *Round 4:*  85.00% White; | *Round 1:* Recruited via mailing lists of public-sector and third-sector organisations, and using social media  *Round 4:* recruited via Prolific; targeted individuals identifying as ‘non-White’ and aged =>50 years |
| Beresford (2023) | 26 | 50.00% male;  34.62% female;  15.39% non-binary/other | Median 39.00  Range 21-64 | 84.62% diagnosed in adulthood;  15.38% diagnosed in childhood | n.r. | Recruited via two diagnostic services, the Autistica Insight Group, and via the research team’s existing networks |
| Crane (2023) | 16 | 31.25% male;  62.50% female;  6.25% gender queer | Mean 49.2  Range 30-79 | Most diagnosed in adulthood; some had a formal autism diagnosis (62.5%) within the past 5 years; some self-identified as autistic (25%); or were exploring whether they were autistic (12.5%) | n.r. | Recruited via mailouts and websites of AutAngel and Autism Matters |
| Crompton (2022) | 12 | 33.33% male; 58.33% female; 8.33% non-binary | Mean 44.92  Range 30-66 | All diagnosed in adulthood; mean age 40.75 | 100% White British/Scottish  /European | Recruited through project website, local autism organisations, social media |
| Scottish Government (2022) | 99 | n.r.^a^ | n.r.^a^ | Most diagnosed in adulthood; 72.72% within past 3 years | n.r.^a^ | Recruited via email invitation by participating services |
| Wigham (2022)^b^ | 343 | 39.94% male; 56.56% female; 3.50% other/did not say | Mean 43.20  Range 19-89 | All diagnosed in adulthood; mean age 40.80 | 92.10% White British | Recruited via the Adult Autism Spectrum Cohort-UK (ASC-UK) |
| Crane (2021) | 16 | 31.25% male; 68.75% female | Mean 44.24  Range 18-71 | Some diagnosed in adulthood | n.r. | Recruited via organisation website and word of mouth; 100% of attendants recruited for first interview; 69% recruited for second |
| Hatton (2021) | 14 | 42.86% male; 57.14% female | Mean 36.00  Range 19-61 | All diagnosed in adulthood recently by NHS service | n.r. | Recruited at point of invitation to group; 70% of attendants from 3 post-diagnostic groups recruited |
| McConkey (2020) | 27 | n.r. | n.r. | Some diagnosed in adulthood, some diagnosed in childhood | n.r. | Recruited via list of service users |
| Westminster Commission (2021) | 248 | 27.30% male; 60.80% female; 10.20% neither; 1.60% did not say | Mean 41.40  Range 17-72 | Most diagnosed in adulthood; mean age of referral 36.5 | 95.90% White British/Irish/other | Recruited via volunteer research databases, autism network, and online sources/social media |
| Beresford (2020) | Quantitative: 308 | 59.74% male; 37.01% female; 2.92% neither | Mean 30.54  Range 17-69 | All diagnosed in adulthood | n.r. | Recruited from NHS diagnostic service |
|  | Qualitative: Subsample of 38 | 50.00% male; 44.74% female; 5.26% neither | Range 17-62 | All diagnosed in adulthood | 86.84% White British/other | Purposive sampling of questionnaire respondents; 71.70% recruited |
| Holtom (2019) | Qualitative: 43 | n.r. | n.r. | n.r. | n.r. | Recruited via IAS |
|  | Quantitative: 124 | n.r. | n.r. | n.r. | n.r. | Questionnaires distributed by service leads and National Autistic Society |
| ARGH (2018) | 41 | n.r.^a^ | n.r.^a^ | n.r. | n.r. | Purposive sampling of HOSS users with survey circulated online and paper |
| Crane (2018) | 10 | 40.00% male; 60.00% female | Mean 42.89  Range 29-59 | Most (90.00%) diagnosed in adulthood; mean age 38.90 | 100% White ethnicity | Stratified random sampling to select subsample of Jones (2014) participants |
| Hickey (2018) | 13 | 76.92% male; 23.08% female | Mean 60.38  Range 51-71 | All diagnosed in adulthood; mean age 53.69 | 100% White British/other | Recruitment via NHS diagnostic service or support and social groups in London; ceased with data saturation |
| Southby (2018) | 14 | 86.71% male; 14.29% female | n.r. | 42.86% diagnosed in adulthood; 21.43% childhood; 35.71% did not say | 100% White ethnicity | Recruited via service email list and face-to-face at drop-in hub |
| Bracher (2014) | 11 | 90.90% male; 9.10% female | Mean 38.70  Range 21-75 | All diagnosed in adulthood by NHS service 6 months prior | n.r. | Purposive sampling from the assessment list; 58% of those invited took part |
| Jones (2014) | 128 | 54.69% male; 45.31% female | Mean 39.20  Range 18-76 | Most diagnosed in adulthood; mean age 34.40 | n.r. | Recruitment via email to organisations for autistic adults; NAS advertisement; support groups |

Note: n.r. = not reported.

^a^Participant characteristics for whole sample provided but no specific breakdown for autistic adult participants.

^b^Authors noted that survey non-responders differed significantly in certain characteristics (gender, age, mental health co-morbidities).
